# Artistic Light and Capturing the Immeasurable

**DOI:** 10.3201/eid1402.0208

**Published:** 2008-02

**Authors:** Polyxeni Potter

**Affiliations:** * Centers for Disease Control and Prevention, Atlanta, Georgia, USA

**Keywords:** Henry Ossawa Tanner, American art, cost-effectiveness, art and science, American expatriates, artistic light, banjo lesion, about the cover

**Figure:**
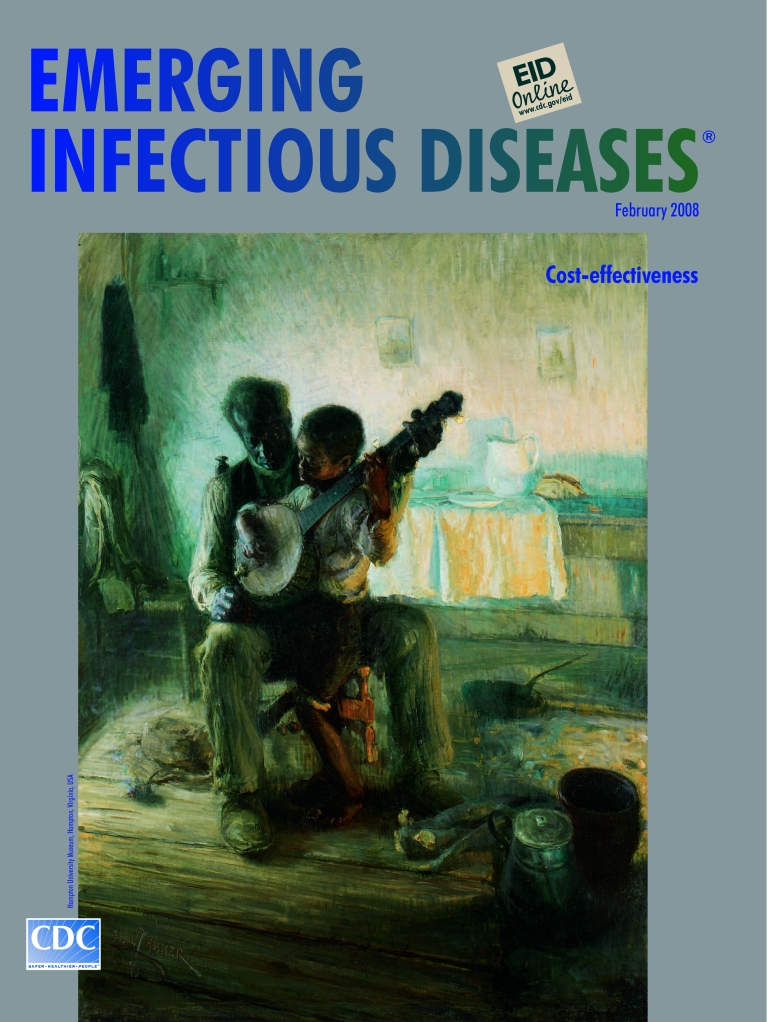
**Henry Ossawa Tanner (1859–1937). The Banjo Lesson (1893).** Oil on canvas (124.46 cm × 90.17 cm). Hampton University Museum, Hampton, Virginia, USA

“After school, I would often go down on Chestnut Street to see the pictures in Earle’s Galleries,” Henry Ossawa Tanner recalled about his early years (*1*). He was especially drawn to the marine subjects of T. Alexander Harrison (1853–1930). “After drinking my fill of these art wonders, I would hurry home and paint what I had seen, and what fun it was” (*1*). When at age 12 or 13, he saw for the first time an artist at work in Philadelphia’s Fairmount Park, he knew his life’s calling.

Tanner was born in Pittsburgh but raised in Philadelphia, the oldest child in a large activist family. His mother, a former slave, escaped through the Underground Railroad. His father was Bishop of the African Methodist Episcopal Church, a denomination formed to protest not dogma but dehumanization and barriers to spiritual expression. “I have no doubt an inheritance of religious feeling,” the artist wrote (*1*). His middle name was derived from Osawatomie, the town in Kansas where abolitionist John Brown started his antislavery campaign.

As often the case with budding artists, the pressure was on Tanner to become successful in a conventional line of work. Nevertheless, he enrolled in the Pennsylvania Academy of Fine Arts, where he studied with Thomas Eakins (*2*). “Get it, get it better, or get it worse,” urged the tough taskmaster and later lifelong friend, “No middle ground of compromise” (*3*). Rigorous training at the academy refined Tanner’s skills as a realist painter. Intolerance cued him in to social realities: “Then he began to assert himself and ... one night his easel was carried out into the middle of Broad Street and, though not painfully crucified, he was firmly tied to it and left there” (*4*).

In 1888, he moved to Atlanta, Georgia, where he opened a photography studio and taught drawing at Clark University. He ventured to nearby North Carolina to photograph and paint the countryside and local folk in their daily activities. But, like most of his contemporaries, Tanner longed to travel abroad to study and experience artistic freedom. With the help of early supporters and patrons, he was able to travel to Europe.

He fell in love with Paris and the “helpful influences” that surrounded him there (*5*). He enrolled in the Académie Julian and studied with Jean-Joseph Benjamin-Constant and Jean-Paul Laurens. “In Paris…no one regards me curiously. I am simply ‘M. Tanner, an American artist’” (*6*). His life there as an expatriate was interrupted only briefly, when a life-threatening bout of typhoid fever sent him back to Philadelphia; then again, when during World War I he moved to England.

Tanner’s work, initially branded by Eakins’ meticulous clarity, gradually acquired its own depth and character. Though mainly in the academic tradition, it incorporated elements of symbolism and impressionism. He was alert and open to new ideas but only as they aligned with his own search for aesthetic and artistic truth. He rejected prescribed alliances in art as much as in his personal life, “I paint the things I see and believe” (*1*). He refused to be categorized or classified.

Light was a central element in Tanner’s paintings and came in shades of Caravaggio or contrasts reminiscent of Rembrandt van Rijn. His palette and loose strokes were suggestive of the impressionists. And whereas his early work reflected all-encompassing interests, his later paintings were exclusively devoted to spiritual themes. But, he wrote, “Religious feeling will not atone for poor art, and vice versa” (*1*). He traveled to the Near East, intrigued by the topography and cultures, which he brought into his biblical scenes in hues of olive and clay, enriching their oriental, mystical quality.

Tanner flourished in France. His work, frequently reviewed in the local press and exhibited in the Salon, was purchased for the national collection at the Musée du Luxembourg. He was named Chevalier of the Legion of Honor. While neither countless awards in France and the United States nor international acclaim could advance his financial situation, the spiritual quality and artistic restraint that came to characterize his work inspired a succeeding generation of artists, among them American favorites Hale Woodruff, Jacob Lawrence, Romare Bearden.

The Banjo Lesson, one of the most famous paintings of the period, was inspired by “Uncle Tim’s Compromise on Christmas,” a short story illustrated by Tanner for Harper’s Young People from a photograph he had staged (*7*). “The only thing in the world that the old man held as a personal possession was his old banjo,” read the story, so his gift to the child had to be shared. But “It was the one thing the little boy counted on as a precious future property, and often, at all hours of the day or evening, old Tim could be seen sitting before the cabin, his arms around the boy….And sometimes, holding the banjo steady, he would invite little Tim to try his tiny hands at picking the strings” (*8*).

Tanner’s empathetic brush captures the intimate moment. Head lowered attentively, the old man surrounds the child, who is at ease and receptive. On the floor lie the implements of their modest life. Faithful to the story, a “long panel of light” bares the cabin’s “smoke-stained wall.” The artist’s masterful technique is rivaled only by the dignity of the scene: gently handing down a prized possession, a music lesson, a life lesson.

In genre as in biblical scenes, Tanner manipulated light to create emotion and drama. In The Banjo Lesson he lights up the interior of a run-down dwelling to reveal what might otherwise be missed: the poor, glowing with humanity and knowledge. These qualities, so clearly expressed in art, can also be seen in the light of science. But because they defy mathematical measurement, they become invisible in cost-effectiveness and other studies, eluding economic analysis and adequate attention.
